# Time to Consider Use of the Sodium-to-Potassium Ratio for Practical Sodium Reduction and Potassium Increase

**DOI:** 10.3390/nu9070700

**Published:** 2017-07-05

**Authors:** Toshiyuki Iwahori, Katsuyuki Miura, Hirotsugu Ueshima

**Affiliations:** 1 Department of Public Health, Shiga University of Medical Science, Seta Tsukinowa-cho, Otsu, Shiga 520-2192, Japan; miura@belle.shiga-med.ac.jp (K.M.); hueshima@belle.shiga-med.ac.jp (H.U.); 2 Research and Development Department, OMRON HEALTHCARE Co., Ltd., 53 Kunotsubo Terada-cho, Muko, Kyoto 617-0002, Japan; 3 Center for Epidemiologic Research in Asia, Shiga University of Medical Science, Seta Tsukinowa-cho, Otsu, Shiga 520-2192, Japan

**Keywords:** sodium-to-potassium ratio, sodium, salt, potassium, dietary intake evaluation, behavior change, self-monitoring, blood pressure, cardiovascular diseases

## Abstract

Pathogenetic studies have demonstrated that the interdependency of sodium and potassium affects blood pressure. Emerging evidences on the sodium-to-potassium ratio show benefits for a reduction in sodium and an increase in potassium compared to sodium and potassium separately. As presently there is no known review, this article examined the practical use of the sodium-to-potassium ratio in daily practice. Epidemiological studies suggest that the urinary sodium-to-potassium ratio may be a superior metric as compared to separate sodium and potassium values for determining the relation to blood pressure and cardiovascular disease risks. Higher correlations and better agreements are seen for the casual urine sodium-to-potassium ratio than for casual urine sodium or potassium alone when compared with the 24-h urine values. Repeated measurements of the casual urine provide reliable estimates of the 7-day 24-h urine value with less bias for the sodium-to-potassium ratio as compared to the common formulas used for estimating the single 24-h urine from the casual urine for sodium and potassium separately. Self-monitoring devices for the urinary sodium-to-potassium ratio measurement makes it possible to provide prompt onsite feedback. Although these devices have been evaluated with a view to support an individual approach for sodium reduction and potassium increase, there has yet to be an accepted recommended guideline for the sodium-to-potassium ratio. This review concludes with a look at the practical use of the sodium-to-potassium ratio for assistance in practical sodium reduction and potassium increase.

## 1. Introduction

Efforts to prevent hypertension and cardiovascular disease (CVD) by dietary improvement have been made for years. In earlier studies, researchers attempted to use dietary intervention as a way of evaluating the effects of a reduced salt intake and an increased fruit and vegetable intakes, while other groups used reduced dietary sodium (Na) combined with the dietary approach to stop hypertension (DASH) diet in order to prevent CVD [1,2,3,4,5]. Since most of these previous interventions showed positive effects and major nutrients affecting blood pressure (BP) became identified in studies [1,2,3,4,5,6,7,8], international recommendations have advised for reducing Na intake and increasing fruit and vegetable intake, i.e., potassium (K) intake [9,10,11,12].

For this review, a literature search was performed in PubMed for publications through 12 June 2017 and from our data. Citations were limited to those published in English. The search referenced the sodium-to-potassium (Na/K) ratio and the cardiovascular variables (blood pressure, hypertension, and cardiovascular disease). Of the 426 unduplicated articles, 318 were excluded based on title (or review of abstract if title was unclear) or publication type. An additional 85 articles were excluded after abstract reviews. After the remaining 23 articles were read in full, 6 were excluded. Ten articles were added by searching reference lists of the remaining 17 articles, resulting in a final inclusion of 27 articles.

## 2. Conventional Dietary Assessment on Sodium and Potassium Separately

Pathogenetic studies have demonstrated that the interdependency of Na and K affects BP [13]. Epidemiological studies have shown that high K mitigates the effect that high Na has on the BP levels, thereby decreasing the risk of CVD [14,15,16,17]. The World Health Organization (WHO) has recommended for years that individuals reduce Na intake and increase K intake; recently, WHO has reiterated its guidelines for Na and K [11,12]. Despite rigorous campaigns to reduce Na and increase K intake, a large gap remains between the recommended and actual intakes of both Na and K [18,19]. Thus, the conventional population approaches have not been able to compensate for this gap [20]. Moreover, the awareness of intakes by individuals remain poor, with subjects who reported practicing reduced-salt diets actually showing salt intake levels similar to those who were not practicing a reduced-salt diet [21]. Thus, an individual approach, such as self-monitoring of Na and K intakes, may aid in a timely achievement of the goals set by the World Health Organization (WHO) of intake levels of less than 2 g of Na per day by 2025 [22].

Previous studies have reported that 80–95% of dietary Na is excreted in the urine [23,24,25,26,27,28]. However, valid estimations of the Na intake can be challenging since random and systematic errors are common [29]. These errors may lead to a paradoxical relationship regarding the Na intake vs. the BP and CVD [30,31]. A prior study reported finding under-collection of the 24-h urine due to limited or no attention to quality control [32]. To reduce both the random error from the high day-to-day variability of Na within an individual and the systematic error due to incomplete urine collection, the use of high quality multiple 24-h urine collection has become the gold standard for estimating the individual daily Na intake [23,24,29,30,33]. Specific approaches that are followed in order to assure the completeness of the 24-h urine collection include asking participants to start and finish the collections in the clinic, and applying rigorous quality control procedures [34,35]. However, collecting repeated high quality 24-h urine is a substantial burden for the study participants. Thus, repeated measurement and high quality measurement for 24-h urine collection in humans are costly, neither easy nor practical to collect.

Single day measurement can be useful for estimating group intake, though it is imprecise to assess as an individual estimate [29,31]. Several suboptimal methods for evaluating Na intake have been suggested, including 24-h urine collected with limited or no attention to quality control, timed overnight urine collection, casual urine collection, 24-h dietary recalls, and the use of food frequency questionnaires. However, the amount of salt used during cooking or added as a table salt is difficult to evaluate when using dietary surveys such as the 24-h dietary recalls and the food frequency questionnaires [36]. One of the major difficulties encountered during public health surveillance studies is the underreporting of the Na intake from the dietary surveys [36]. Although the measurement of casual urine collections is much easier to perform than the other methods, the most commonly used formulas for estimating the 24-h urine Na excretions have a problematic bias [37,38,39]. Even though these formulas contain less bias for the population mean salt intake level, overestimations in the low salt ranges and underestimations in the high salt ranges are the major factors that can lead to irrelevant conclusions in association studies that examine salt intake and CVD [31,40,41], e.g., findings from PURE (Prospective Urban Rural Epidemiological) study [42,43]. Furthermore, since these formulas depend on other parameters such as body weight and creatinine levels, this makes it uneasy for the patients to undertake self-monitoring [37,38,39]. Therefore, a more reliable and easier method for assessing the Na levels is required.

Similarly, the amount of K excreted in the 24-h urine is less than that of Na, as relatively more K is excreted in the stool. A previous study reported finding a high correlation between the K intake and the 24-h urinary K excretion [23]. Thus, K excreted in the 24-h urine provides a good estimate of the dietary K intake [23,33,44]. Other reports have also stated that 63–77% of the dietary K is reflected in the urinary K [23,25,26,27]. The amount of K can also be evaluated by use of 24-h dietary recalls [33,36]. A high correlation is also reported between the actual K intake and the K intake estimated by the 24-h dietary recall [23]. However, obtaining either a 24-h urine collection or a 24-h dietary recall is a major difficulty for public health surveillance studies, as repeated collections are needed in order to reliably estimate the intakes [23,29,44]. Although the measurement of casual urine is much easier than other methods, the formulas for estimating the 24-h urine K excretions are less commonly used compared to those for the evaluation of the 24-h urine Na excretions [37].

## 3. The Na/K Ratio: A Surrogate Index for Higher Na Intake and Lower K Intake

A high urinary Na/K ratio is an indicator of a higher Na intake and a lower K intake [45,46,47]. These ratios are easier to measure due to the independence of the urine collection or the creatinine measurements. The present review examines the practical use of the Na/K ratio, which is a surrogate index of the dietary Na reduction and K increase, for supporting dietary change.

### 3.1. Epidemiological Findings for the Na/K Ratio

Most epidemiological studies have shown that the Na/K ratio in the 24-h urine is cross-sectionally associated with BP [6,7,8,48,49,50,51,52,53,54,55,56,57,58,59,60]. In addition, the Na/K ratio has been reported to be a superior metric vs. either Na or K alone in relation to BP [6,7,8,55]. Use of the Na/K ratio is resistant to systematic errors related to incomplete urine collection or underreporting of intake from 24-h recalls. These may contribute to diminish the paradoxical results often seen in association studies.

Findings from the large multicenter international cooperative study on salt, other factors, and blood pressure (INTERSALT) population study demonstrated that the change in the urinary Na/K molar ratio from 3.09 to 1.00 delivers 3.36 mmHg of estimated reduction in the population systolic BP (Table 1) [6,7]. The estimated reduction was larger for the Na/K ratio compared to when the Na and K were analyzed separately [6,7]. Reductions of 3–5 mmHg in the population BP has been estimated to lead to reductions of approximately 8–14% in the mortality of stroke, 5–9% in the mortality of coronary heart disease, and 4–7% in the total mortality [61]. Thus, the reduction of the Na/K ratio is indirectly effective for the prevention of CVD. However, the TOHP (Trials of Hypertension Prevention) study reported finding a direct association between the urinary Na/K ratio and the CVD [62,63,64]. Comparison of the TOHP study data between the lowest (<2) and the highest (≥4) urinary Na/K ratio category determined the hazard ratio (HR) for all-cause mortality was 0.75 (95% confidence interval (CI): 0.47 to 1.20) [63]. There was also a linear relationship observed for the mortality, with an HR of 1.13 per unit increase in the urinary Na/K ratio (*p* = 0.035) [63]. Furthermore, the 10–15 years of post-trial follow-up for the TOHP study showed that a higher Na/K ratio was associated with an increased risk of later CVD, which was greater than that for either Na or K alone [62]. The TOHP follow-up study also found there was a significant trend for the CVD risk regarding the gender-specific quartiles of the Na/K ratio (HR = 1.00, 0.84, 1.18, and 1.50, *p*-trend = 0.04) [62]. In addition, there was also a statistically significant linear association between the urinary Na/K ratio and the risk of CVD, with an HR of 1.24 per unit (95% CI = 1.05–1.46, *p* = 0.012) [62].

Association studies that utilized the dietary recall and dietary record methods from the national health and nutrition examination survey (NHANES) III and the national integrated project for prospective observation of non-communicable disease and its trends in the aged (NIPPON DATA80) cohort study also reported similar findings on Na/K ratio [65,66]. The report from the NHANES III cohort studies showed that the HR for the highest vs. the lowest quartile of the dietary Na/K ratios were 1.46 (95% CI: 1.27–1.67) for all-cause mortality, 1.46 (95% CI: 1.11–1.92) for cardiovascular diseases, and 2.15 (95% CI: 1.48–3.12) for ischemic heart disease [65]. Similarly, the findings from the NIPPON DATA80 cohort study showed that the HR for the highest quartile vs. the lowest quartile of the dietary Na/K ratios (mean dietary Na/K molar ratio: 2.72 vs. 1.25) were 1.43 (95% CI: 1.17–1.76) for stroke, 1.39 (95% CI: 1.20–1.61) for cardiovascular diseases, and 1.16 (95% CI: 1.06–1.27) for all-cause mortality [66].

### 3.2. Casual Urine Estimates for the 24-h Urine Value for The Na/K Ratio

**Figure 1:**
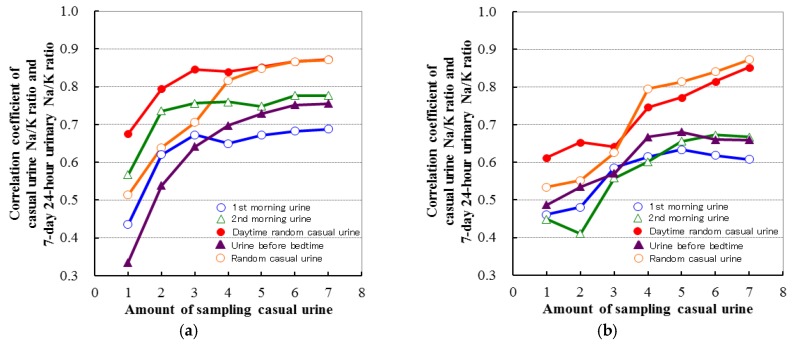
Correlation specification between numbers of repeated casual urine sampling and 7-day 24-h urine of sodium-to-potassium (Na/K) ratio in normotensive and hypertensive individuals (made from data of [67,68]). (**a**) Normotensive individuals; (**b**) Hypertensive individuals.

**Figure 2:**
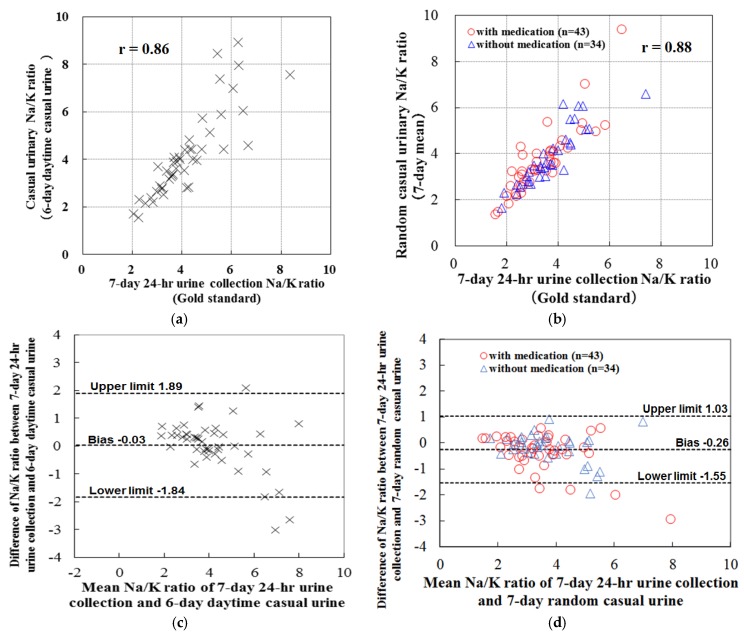
Plots of Na/K ratio of casual urine vs. 24-h urine, and Bland–Altman plots in normotensive and hypertensive individuals (made from data of [67,68]). (**a**) Normotensive individuals (scattered plots, *n* = 45); (**b**) Hypertensive individuals (scattered plots, *n* = 77); (**c**) Normotensive individuals (Bland-Altman plots, *n* = 45); (**d**) Hypertensive individuals (Bland-Altman plots, *n* = 77).

Higher correlations are seen for the casual Na/K ratio vs. the individual casual Na or K when compared with the 24-h urine values [45]. Furthermore, the population mean of the 1-day 24-h urine Na/K ratio can be estimated from the population mean of the single casual urine Na/K ratio within the diverse worldwide population [45]. High correlation (*r* = 0.80–0.88) and good agreement are seen between the mean value of repeated casual urine Na/K ratio and the 7-day 24-h urine Na/K ratio in Japanese normotensive individuals and Japanese hypertensive individuals (who were primarily taking calcium channel blockers (CCBs), angiotensin 2 receptor blockers (ARBs), or both CCBs and ARBs) (Figure 1 and Figure 2) [67,68]. Evidences showed that the association between casual and 24-h urine Na/K ratio were robust to use of ARB, CCB, and thiazide diuretics [45,68]. The correlation and agreement quality of mean Na/K ratio of 4–7 repeated measurements of casual urine with 7-day 24-*h* urine Na/K ratio were similar to that of 1–2 day 24-*h* urine Na/K ratio with 7-day 24-*h* urine Na/K ratio [67,68]. There are several benefits for using the casual urine estimate for the 24-*h* urine value used to determine the Na/K ratio. These benefits include the independence of the urine volume, creatinine excretion, and body weight, the fact that repeated random sampling minimizes the systemic error caused by diurnal variation and day-to-day variation in the Na/K ratio [69], that there is less bias observed in the low to high salt range, and that the gold standard set for the casual urine estimate is the 7-day 24-*h* urine when the single day 24-*h* urine value is used for Na and K separately [67,68]. Therefore, the repeated casual urine Na/K ratio measurement may be one of the most reliable individual estimates for assessing intakes involved with Na reductions and K increases in normotensive and hypertensive individuals. Through the use of these estimates, this might make it easier to screen individuals who need to make dietary lifestyle modifications in addition to improving the awareness of an individual’s dietary levels. However, the association between the repeated casual urine Na/K ratio and the 24-*h* urine Na/K ratio has yet to be examined in the elderly (ages 70 or older) and in individuals with chronic kidney disease or diabetes, or in subjects being administered several different types of anti-hypertensive medication therapies (loop diuretics, beta-blockers, angiotensin converting enzyme inhibitors, alpha-blockers, central agonists, or combinations of these different drugs). Further validations will need to be performed in all of these areas.

### 3.3. Target Level of Na/K Ratio

Findings from the INTERSALT study demonstrated that the mean 24-h urine Na/K molar ratio ranged from 0.01 (Yanomamo, Brazil) to 7.58 (Tianjin, China) [6,45]. Mean Na/K ratios in Asian and Western populations were approximately 5 and 3, respectively [6,45]. Considering the ratio calculated by population mean 24-h urine Na/K molar ratio and population mean 24-h urine salt (NaCl) (g/day) may give a rough estimate for predicting NaCl intake from Na/K ratio; population mean 24-h urine Na/K molar ratio was 3.24 and population mean salt intake estimated by 24-h urine was 9.1 (g/day) thus the mean value of the ratio was calculated as 2.8 (ranged from 1.3 to 5.3 among 52 populations (mostly 2 to 4)) in 10,079 individuals among 32 countries in INTERSALT study [45]. Thus, a rough estimate of population mean salt intake (g/day) may be given by approximately 2–4 times of population mean 24-h urinary Na/K molar ratio in the general populations. Furthermore, Yatabe et al. demonstrated that the daily population means of the urinary Na/K ratios traced dietary salt intake within 2–3 days during an experimental feeding study, with 4.2 for the unrestricted diet, 1.1 for the low-salt 3 g/day diet, and 6.6 for the high-salt 20 g/day diet periods [70,71]. Currently, there is no generally accepted recommended guideline for the Na/K ratio. Based on the findings from the INTERSALT study, Stamler et al. recommended that an urinary Na/K molar ratio of 1.0 be used for the target level [7]. Reports from WHO suggested that achieving the guidelines for both the Na and K intakes would yield an Na/K molar ratio of approximately 1.00 [11,12]. Cook et al. also reported that Na/K molar ratios between 1 and 2 exhibited the lowest CVD risk [62]. Therefore, although urinary Na/K molar ratios less than 1 are preferable, ratios less than 2 might be an interim suboptimal goal for most people trying to lower their BP and reduce the CVD risk.

## 4. Self-Monitoring of the Urinary Na/K Ratio

Reducing the Na/K ratio is essential for preventing hypertension and CVDs prior to clinical onset [6,7,8,48,49,50,51,52,53,54,55,56,57,58,59,60]. Urinary Na/K ratios can be measured by urinary Na and K concentrations, or by performing measurements in a central inspection lab. One of the methods for sampling urine specimens and identifying an individual’s Na/K ratio levels is to collect the casual urine specimens using small spout containers, followed by measuring the mean values at the clinician’s office. However, due to the time required for the collection, delivery, and measurement, this method may not provide prompt enough feedback for the patients to make further dietary modifications.

Nowadays, urinary Na/K ratio can be measured by a portable self-monitoring device (HEU-001-F, OMRON Healthcare Co., Muko, Japan). This device measures urinary Na/K ratio by the ion electrode method and displays the result within one minute. Since this device provides prompt onsite feedback in personal use, it became evaluated in randomized control trial with a view to support an individual approach for Na reduction and K increase [72]. With regard to the individual approach, it is important that there is a balance between lower effort and financial burden in order to achieve an effective intervention. A recent study that examined this self-monitoring device reported finding a trend for larger reductions in the urinary Na/K ratio in a self-monitoring group under a pure self-management setting. When the baseline urinary Na/K molar ratio level was approximately 3.7, the reductions in the urinary Na/K molar ratio were 0.55 in the intervention group and 0.06 in the control group [72]. However, the intervention effect size was limited due to the lack of an effective education program for reducing the Na/K ratio. Since self-monitoring devices are different from therapeutic devices, these monitoring devices may be powerless unless the subjects also take part in a practical dietary program. Thus, it would be expected that there would be a much larger reduction when these devices are combined with a useful education program. Furthermore, if individuals are able to acquire basic knowledge and skills for Na reduction and K increase, this could be one of the key factors for improving confidence and enhancing motivation for dietary improvements. Therefore, providing feedback to individuals at appropriate times might also help to support the effectiveness of these education programs.

## 5. Implications for Prevention and Treatment

Na surfeit and K deficit are of worldwide concern for hypertension, cardiovascular diseases, and non-communicable diseases. Measurement of the Na/K ratio is much easier to obtain than trying to perform Na and K measurements separately. Thus, an awareness of dietary levels through the use of this index may lead to improvements in an individual’s ratio. The individual estimate of the 24-h urinary Na/K ratio that can be obtained by the repeated casual urine Na/K ratio may be useful in detecting individuals who need an easy dietary lifestyle modification during the prevention stage. For the treatment stage, self-monitoring devices may increase patient awareness of their dietary level and help to maintain appropriate levels since evidences showed that the association between casual and 24-h urine Na/K ratio were robust to class of use of anti-hypertensive medication [45,68]. The urinary Na/K ratio level objectively reflects a patient’s recent dietary status [70,71]. Thus, information on the urinary Na/K ratio is essential when trying to determine the types and doses of anti-hypertensive medication therapy. However, dietary K restriction is advised for those with impaired kidney function in advanced stage of chronic kidney disease (CKD). Therefore, it is reasonable to infer that the Na/K ratio may be safe and beneficial index to reduce Na and increase K intake for individuals who has not reached to the advanced stage of CKD.

Use of an individual approach in conjunction with an effective education program for lowering the Na/K ratio and self-monitoring tools for the urinary Na/K ratio may help improve individual’s Na and K intake levels and support their attempts at a lifestyle modification. In addition, utilizing a combination of the conventional population approach and the individual approach focused on the Na/K ratio may help to minimize the gap between the actual and ideal ratio levels. Therefore, implementation of the use of the Na/K ratio as a way for practical Na reduction and K increase may be a key factor for reaching the 2025 goal set by WHO of achieving intake levels less than 2 g of Na per day [22].

## Figures and Tables

**Table 1:** Predicted differences in population mean systolic blood pressure with lifestyle variables.

Lifestyle Variable	Present Level	Improved Level	Predicted Difference
Na	170 mmol *	70 mmol	−2.17 mmHg
K	55 mmol *	70 mmol	−0.67 mmHg
Na/K	3.09 *	1.00	−3.36 mmHg
BMI	25.0 *	23.0	−1.55 mmHg
High Alcohol	≥300 mL/week ^‡^	1–299 mL/week ^‡^	−2.81 mmHg
Improved levels of both Na/K and BMI	-	-	−4.91 mmHg
Expected difference if heavy drinkers also reduced alcohol	-	-	−5.33 mmHg

* Approximate median level found in INTERSALT. ^‡^ Reported by 15% of respondents. Na: sodium; K: potassium; Na/K: sodium-to-potassium ratio; BMI: body mass index. INTERSALT: the international cooperative study on salt, other factors, and blood pressure. Tables created based on results from [7].
